# Bringing Young People, Health and Social Care Professionals, Transition Champions and Policymakers Together Through Hybrid Methods of Participation: Creating a Space for Shared Understanding of What Is Required to Improve Young People's Healthcare Transition

**DOI:** 10.1111/hex.70136

**Published:** 2025-01-30

**Authors:** Louise Porter, Faith Gibson, Susie Aldiss, Sue Morgan, Alexandra Stanton, Stella Carney, Aishah Farooq, Isabel Barlow, Haris Sultan, Pippa Sipanoun

**Affiliations:** ^1^ The Burdett National Transition Nursing Network England; ^2^ Leeds Teaching Hospitals NHS Trust Leeds UK; ^3^ School of Health Sciences University of Surrey Guildford UK; ^4^ The Centre for Outcomes and Experience Research in Children's Health, Illness and Disability, Great Ormond Street Hospital London UK; ^5^ UCL Faculty of Population Health Sciences, UCL Great Ormond Street Institute of Child Health London UK; ^6^ Somerset Healthcare Foundation Trust Somerset UK; ^7^ CYP Transformation Programme Board at NHS England UK; ^8^ NHS Assembly Member at NHS England UK; ^9^ NHS WEST Yorkshire Integrated Care Board UK

**Keywords:** collaboration, healthcare professionals, healthcare transition, hybrid methods, long‐term conditions, policymakers, young people

## Abstract

**Introduction:**

A multi‐stakeholder conference was held in 2023, celebrating the achievements of the Burdett National Transition Nursing Network (BNTNN). The BNTNN had been implemented across England in 2020 to map the current state of young people's healthcare transition into adult services across England, and work with key stakeholders to coach them through making sustainable quality improvements to young people's transition services. This work was funded by the Burdett Trust for Nursing, following the success of an exemplar Model for Quality Improvement (QI) for Transition, which had been developed at a Teaching Hospital in England. The BNTNN consisted of a National Lead Nurse, four Regional Nurse Advisors based in host organisations in the four main regions of England, and an Expert Advisor for the care of young people. A research team was appointed to evaluate the impact of the BNTNN, leading the National Transition Evaluation Study. The BNTNN Lead Nurse worked in partnership with NHS England to provide national solutions to high‐level barriers to the implementation of transition pathways.

**Methods:**

Young people with long‐term conditions have participated and engaged with the BNTNN since its inception, throughout the QI project and research through membership to the Transition Advisory Group. Young people, professionals, staff members and policymakers were included in our hybrid conference in March 2023. The BNTNN and research team brought these groups together to share learning from the 3‐year project, celebrating and showcasing achievements in each region as a result of the expert advice and support from the network. Young people contributed their experiences of transition journeys into adult services, and policymakers reflected upon national developments. Provider organisations from each region showcased their transition transformation journeys, sharing successes and challenges encountered during the QI process. The research team provided an update, and was responsible for capturing content and discussions on the day.

**Results:**

With 405 attendees, the conference provided peer support and guidance, and enabled connections between young people, health and social care professionals, transition champions and policymakers. The primary aim was to forge long‐lasting collaborations for the benefit of improving health services and outcomes for young people.

**Conclusion:**

In this article we highlight how it is possible to bring key stakeholders together through hybrid methods of participation, and how this enabled a shared understanding and a combined commitment to progress young people's transition services for the future.

**Patient or Public Contribution:**

Young people who are experts by experience have been involved throughout this 3‐year project and in the ongoing evaluation. Here we highlight the importance of involving young people, professionals, staff members and policymakers when creating a space for shared understanding of what is required to improve services for young people transitioning into adult healthcare services.

**Trial Registration:**

NCT05867745 [ClinicalTrials.gov].

## 1 Introduction

A multi‐stakeholder conference was held in 2023, celebrating the achievements of the Burdett National Transition Nursing Network (BNTNN). The BNTNN had been implemented across England in 2020 to map the current state of young people's healthcare transition into adult services across England and work with key stakeholders to coach them through making sustainable quality improvement (QI) approaches to young people's transition services.

Getting young people's healthcare transition right is crucial because young people with long‐term health conditions face challenges as they deal with complex and important changes in their need and access to healthcare during this process of transitioning from children's into adult services [1]. For many, healthcare transition does not meet their medical, psychosocial, emotional, educational and vocational needs, leading to young people experiencing poorer health outcomes [1, 2, 3].

**Figure 1:**
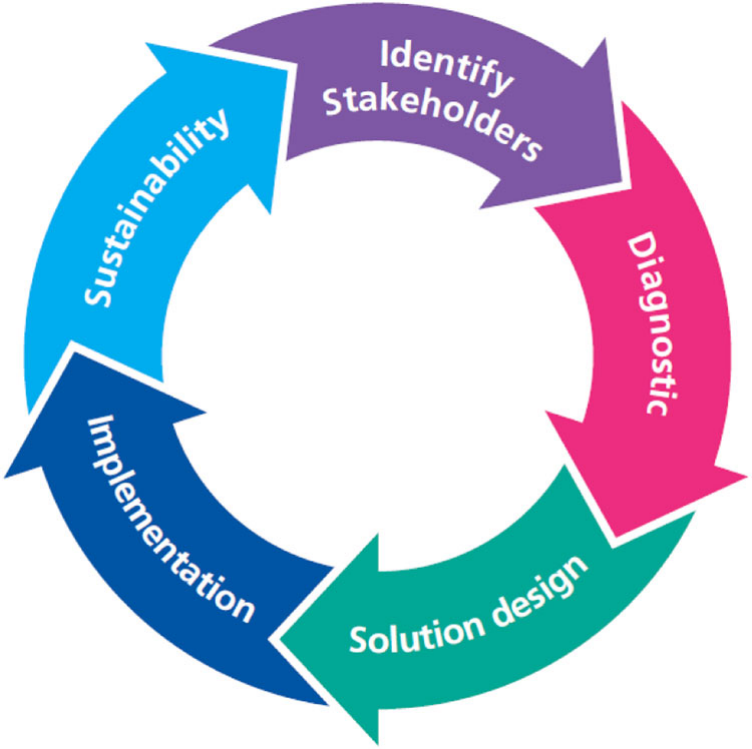
Model for Improvement for Transition.

**Figure 2:**
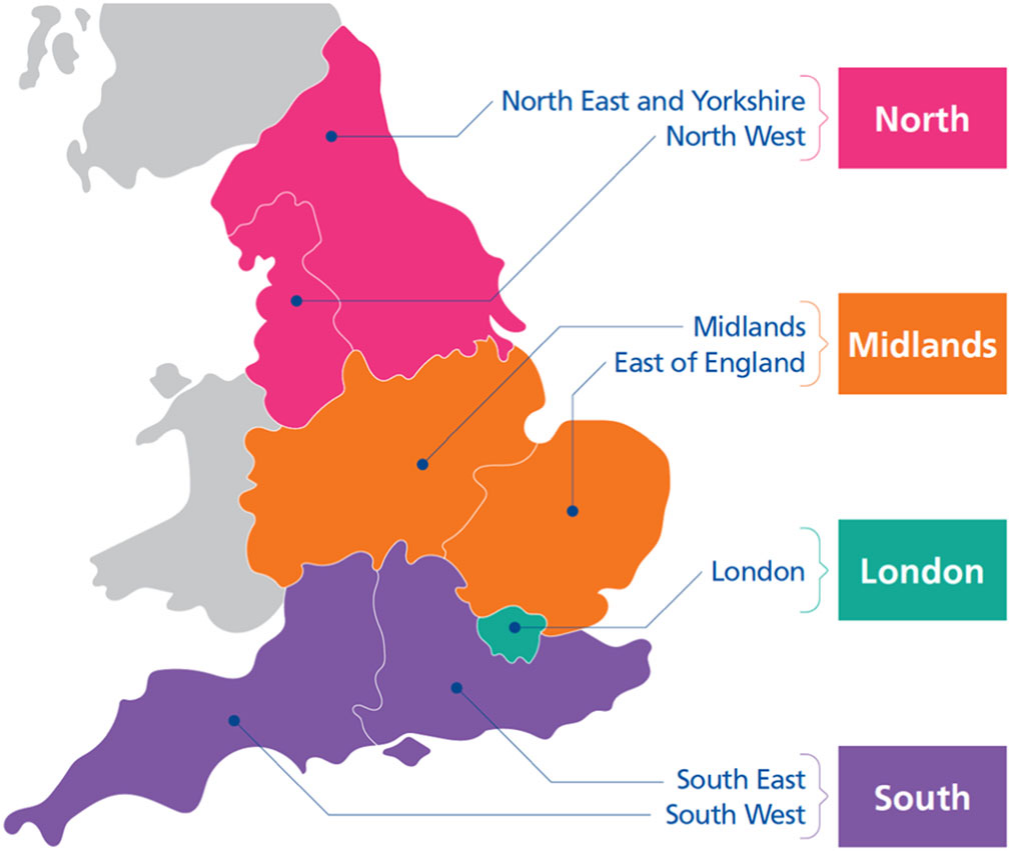
National Transition Nursing Network implementation across England.

To enable effective, planned and coordinated processes for young people's transition, an exemplar Model for QI for Transition was developed at a large Teaching Hospital in England. Development was underpinned by QI methods that have their roots in the motor vehicle manufacturing industry [4, 5], and methods such as ‘Lean’ focus on ensuring optimal flow, minimal mistakes and waste [6]. These methods informed the Model for QI for transition. The model was made specific to transition through inclusion of pertinent transition‐related processes and documents within each section (Figure 1), which was then utilised by the BNTNN for national implementation following the model's success. This work was funded by the Burdett Trust for Nursing. The BNTNN consisted of a National Lead Nurse, four Regional Nurse Advisors based in host organisations in the four main regions of England (Figure 2) and an Expert Advisor for the care of young people. A research team was appointed to evaluate the impact of the BNTNN, leading the National Transition Evaluation Study. The Lead Nurse worked in partnership with NHS England to provide national solutions to high‐level barriers to implementation of transition pathways.

Young people with long‐term conditions have participated and engaged with BNTNN since its inception, throughout the QI project and research, through membership of the Transition Advisory Group, promoting the principles of patient and public involvement and engagement [7]. The BNTNN and research team brought young people, professionals, staff members and policymakers together in a hybrid conference in March 2023, to share learning so far from the 3‐year project, celebrating and showcasing achievements in each region as a result of the expert advice and support from the BNTNN. Young people contributed their experiences of transition journeys into adult services and provider organisations from each region showcased their transition transformation journeys, sharing successes and challenges encountered during the QI process. Policymakers reflected upon national developments, and the research team provided updates and was responsible for capturing content and discussions on the day.

This conference was an opportunity to share good practice and celebrate the work achieved by the BNTNN, and was important for information sharing and gathering, aimed at building lasting network connections with attendees from across England: to sustain the transition work that had already been achieved, and to reinforce the transition community of practice ethos where everyone is a teacher, and everyone is a learner. Presentations were delivered by members of the network highlighting results from the network's QI projects. Visible across all presentations was the involvement of young people, health and social care professionals, transition champions and policymakers known to influence change at organisational, regional and national level. This provided networking opportunities to further develop connections and collaboration for transition improvement work to continue and progress.

The conference was attended by professionals from children's and adult services with representatives from all healthcare disciplines, across acute, primary, secondary, tertiary and palliative care. Four young people with lived experience of healthcare transition attended, two of whom shared their views after attending the NHS England Youth Summit transition workshop event in February 2023. ‘Transition Champions’ included transition leads and those involved in QI work, such as from charities, local authority and social care staff, including youth workers, administrative staff, parent/carer support groups, special educational needs and disability staff, alongside policy leads, national and regional leads from NHS England. We report here feedback and learning from the event.

## 2 Methods and Analysis

In‐person conferences provide a traditional platform for disseminating the latest research findings and for networking. The pandemic forced us to think differently, more creatively, about how we might achieve the same outcome from an online/hybrid event. Cost, accessibility and safety at an event, known challenges for conference attendance, are minimised with an online event. Dissemination can be efficient online, but facilitating opportunities for networking can be more challenging. For us it was important to bring together the range of healthcare transition stakeholders, young people, health and social care professionals, and policymakers, to share our learning, therefore a hybrid event was selected. Key to our planning was tailoring content, maximising reach, enabling networking, making use of multimedia and facilitating the meaningful contribution of young people.

We ran this event, with the support of conference specialist information technology, and a Project Support Assistant (A.S.). Content was planned with all members of the network, enabling and empowering network members to present their progress in setting up and/or making changes to young people's transition services, and any barriers or facilitators they experienced. A face‐to‐face panel discussion with policymakers was also included, and online attendees were able to join the debate, bringing together traditional and online conference methods to work for everyone.

We utilised the interactive online platform Mentimeter to establish the attendees' level of engagement and commitment to change. Two members of the evaluation team (P.S., S.A.) analysed information gathered from the Mentimeter using qualitative content analysis [8]. Responses were exported from Mentimeter into Excel. The researchers read through the responses to familiarise themselves with the data. Initial coding was undertaken, using colour highlighting within the spreadsheet to identify responses on similar topics. Responses were arranged by colour groupings/codes. These groupings formed the initial themes. The groupings and themes were refined and finalised through discussion, until a consensus was reached. Themes were discussed and finalised, and any differences in themes from Question 1 and Question 3 (a follow‐up to Question 1) were highlighted.

## 3 Results

A total of 405 delegates (370 people online and 35 in person) attended the conference.

### 3.1 Mentimeter Figures and Feedback

Question 1 ‐ Professional Commitment: What commitment will you make to progressing young people's transition for the future?

Ninety‐six people participated in Question 1, with 169 responses (people were able to submit more than one answer via Mentimeter). Seven themes were identified (Table 1). Three responses were not placed in a theme as they were unclear.

**Table 1:** Themes and example responses from Question 1.

Theme	Illustrative responses
Collaboration between young people, parents and professionals	Commit to starting a good and cohesive collaboration between children's and adult services. Improved communication and shared working between children's teams, adult teams and YP. Pushing forwards the joined up working that will improve the process. To collaborate not only with young people and their families but with professionals also involved in their care.
Young person‐centred transition	Ask who is important to their transition journey. Ensuring the young person is involved throughout the transition process. Making the transition clinics more about the young person. To ensure my approach is person centred, to hear them and be their advocate. Listening to the wishes and feelings of the young person.
Care coordination across all sectors	Joined up working with community services and elective hospitals for young people with LD and long‐term health needs. I will aim to create a transition clinic letter for GP letters that clearly spells out their accountability towards allergy patients that return to GP care as adult allergy service is limited. To continue to explore how the hospice sector can not only improve hospice transition but support our young people in all their transitions. Promote the need for engaging across community/primary care/voluntary sector and not relying purely on acute services to ensure a smooth transition.
Training, education and support for professionals	Raise the profile of transition and young people services in our trust and why this is SO important. Improving knowledge of the needs of the young person during the transition process for my team, which includes engagement strategies, awareness of key periods of handovers, etc. Continue to provide education around the ‘how to’ of transition. Setting up peer‐to‐peer support networks. Sharing resources and good practice.
Information, education and support for young people and families	Committing to learning more about how adult services are structured and provided so I can be a better support to CYP and their families when planning the transition. Try to set up meet and greet with patients who have been through transition so they can have support and share experiences. Building confidence in young people. Building the Youth Service so every young person with a long‐term health condition has the support of a Youth Worker. Work more closely with parents/carers of our complex needs young people who are non‐verbal/limited communication with profound LD.
Improving the transition process	Start transition earlier. Set up a clear pathway for the young person and their support network to provide reliable information on who will take over their care and work towards achieving joint handover clinics. Continue to push my Trust and senior leaders to improve our transition service. Increase the transition workforce in our hospice. Ensure that all young people on our caseload have a transition passport.
Youth involvement/patient and public involvement and engagement (PPIE)	Undertake further work with CYP and families to understand more about what works best. Engage YP to influence change on what they want transition to look like for them, and use their voice to steer key holders to make changes! Ask YP what they want to see and how transition can be improved locally. Utilise youth forum and create more opportunities for the young people involved. I will ask some young people if they would be willing to speak to our adult colleagues about their experiences and what things would have made it easier for them.

Abbreviations: GP, general practitioner; LD, learning disability; YP, young people.

### 3.2 Collaboration Between Young People, Parents and Professionals

Commitments were listed which involved improving collaboration and relationships between child and adult teams, along with more joined‐up working with other services. Collaboration included not only working with colleagues but also with young people and parents.

### 3.3 Young Person‐Centred Transition

Ensuring the transition process is focused on the young person, that their views are heard, and care meets their individual needs. Specific commitments involved listening to and advocating for young people. Involving parents in discussions alongside young people was also mentioned.

### 3.4 Care Coordination Across All Sectors

Commitment to involvement and collaboration between different services, including primary care, community services, the voluntary sector and hospices. This was in reference to young people with complex needs or learning disabilities who use multiple services or where there was not an adult service for the young person to be transferred into (such as for young people with allergies) and where the GP would lead on their care going forward.

### 3.5 Training, Education and Support for Professionals

Commitment to education for staff about transition, building networks to share information, good practice and provide support. Responses referred to raising the profile of transition and its importance among professionals.

### 3.6 Information, Education and Support for Young People and Families

Commitment to increasing the support available for young people and families during the transition process in a way that considers their individual needs. Suggestions of how this support could be provided included youth workers and peer support events where they could share their experiences. Improving young people's knowledge of their condition, and provision of information about the transition process were mentioned – involving parents as well, specifically highlighted was the involvement of parents of young people with learning disabilities/complex needs.

### 3.7 Improving the Transition Process

This theme had the highest number of responses. Professionals mentioned several ways they wanted to work towards improving transition. This included making major changes within their service such as the use of transition documentation and hospital passports, setting up transition clinics, implementing transition pathways, joint handovers between children's and adults' care providers, and increasing the transition workforce. Some responses indicated that these changes had already begun and expressed a commitment to continuing them.

### 3.8 Youth Involvement/PPIE

Many respondents wrote about their commitment to involving young people in service development, finding out about their experiences and what they want transition to look like. Setting up a youth forum or engaging with existing youth forums were identified as ways to hear from young people.

Question 2 ‐ **What one aspect of transition needs to change to have maximum impact for young people going forward?**

**Figure 3:**
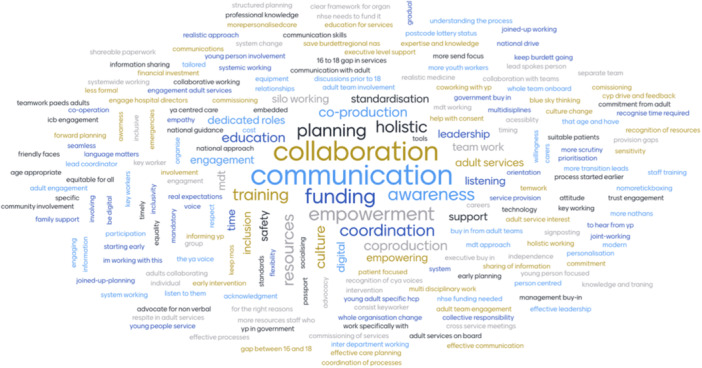
Responses from Question 2.

For this question, we created a Mentimeter word cloud of responses (Figure 3). Eighty‐five people responded to this question with 301 answers.

The depth and breadth of the concepts raised in this word cloud demonstrate the complexity of getting young people's transition into adult services right. Within a word cloud, the larger the text, the more that response had been entered. Communication, collaboration, funding and resources, awareness, empowerment, coordination and coproduction are all prominent topics. Planning, training and education and holistic, inclusion, safety, time, support, standardisation, culture and having dedicated roles within transition also feature as key issues. These responses echo the themes represented in Question 1, highlighting the audiences' awareness and commitment towards driving positive change within young people's transition.

Question 3 **‐ Professional Commitment (recap): Has the commitment you stated this morning changed following today's sessions? How and why? What are your next steps?**

Forty‐one people participated in Question 3, which was posed at the end of the conference. There were 58 responses, from which 7 themes were identified. Five themes were concordant with themes identified to Question 1; two new themes were identified (Table 2).

**Table 2:** Themes and example responses from Question 3.

Theme	Illustrative responses
Collaboration between young people, parents and professionals	Yes – thinking about more collaborative transition planning/pathways with others (most importantly starting it with CYP and families – from the beginning). To explore establishing/re‐stablishing a transition network. Networking to ensure good communication and practice. Not changed, explore more joint working with adult teams for complex needs YP.
Young person‐centred transition	No – hasn't changed, ask more questions around what young people want to get out of the experience of transition. Always offer to be seen without parent. Ensure I talk more to patients than their parents. Not to dismiss any questions asked by the patients. Always listen to young people's voices and what they want.
Training, education and support for professionals	The commitment is still the same. I will share with my team the resources available. I have an upcoming youth focus group planned but would like to access ‘Me First’ training too. Still want to set up peer‐to‐peer support. Education to those stakeholders surrounding YP.
Improving the transition process	More funding required for adult health professionals to be able to help and transition young adults effectively. Refreshed the need to evaluate and audit current transition practice. As a result of conference today have emailed Trust CNO to raise transition as an issue within Trust, hopefully this will start talks around transition within Trust management. Recognise team successes: face‐to‐face joint‐led nurse and consultant transition clinics and use of ready/steady/go transition framework.
Youth involvement/PPIE	Starting with finding out what our young people would like the service to look like. To include CYP voice throughout transition planning. Giving the children and young people an opportunity for their voices to be heard. Feel more passionate about involving young people in further development of my service. Really focus on co‐creation.
Feeling a sense of responsibility	Not changed but provided more evidence for reasons why it is important. Gives me reassurance that our transition programme is good but still has some gaps to fill. Feel overwhelmed with the responsibility to try and get this right for these young people.
Increased commitment and motivation; being more proactive	Commitment remains the same but renewed sense of vigour. Not changed but I feel more motivated and more equipped. Not changed! More determined than ever to keep improving! Even more aware of the importance of transition and getting the right processes and discussions in place. Need to be more proactive and feed into the work that is happening. More committed to making transition important to everyone! Feel empowered and invigorated to get out there and make a difference! No, it's emphasised the importance of pledging my commitment.

Abbreviations: CNO, Chief Nursing Officer; CYP, children and young people; YP, young people.

Two themes that were derived from the responses to Question 1 were not apparent in this follow‐up question: care coordination across all sectors and information, education and support for young people and families.

### 3.9 Collaboration Between Young People, Parents and Professionals

Responses focused on future collaborations that could be forged that would push forward the agenda of young people's transition, for example, establishing a transition network, improved communication and joint working, and thinking about more collaborative transition planning/pathways.

### 3.10 Young Person‐Centred Transition

In addition to asking young people who is important to their transition journey, as raised in Question 1, finding out what young people want to get out of the experience of transition was raised in this follow‐up question. Responses highlighted the importance of talking to, listening to, and involving young people and their parents, as well as the need to always offer the young person the opportunity to be seen without a parent.

### 3.11 Training, Education and Support for Professionals

Being able to access training that would support the respondents' ability to work with young people, for example, the ‘Me First’ training (training on communicating with children and young people, www.mefirst.org.uk/training/), was highlighted, as well as support and education for those working with young people and their families.

### 3.12 Improving the Transition Process

The need to have Trust management and executive buy‐in to progress young people's healthcare transition was raised again in this follow‐up question, with actions resulting directly from the conference. Topics additional to those raised in Question 1 were also highlighted, such as, the need to evaluate and audit current transition practice and the need for more funding.

### 3.13 Youth Involvement/PPIE

Respondents were dedicated to involving young people throughout the transition process, with a focus on co‐creation, and emphasis on the need to ensure the young person's voice is heard.

### 3.14 Feeling a Sense of Responsibility

These responses highlighted awareness of the work that needs to take place and the responsibility they felt in getting transition right for young people. One participant reported feeling ‘overwhelmed', emphasising the importance of ongoing networks of support.

### 3.15 Increased Commitment and Motivation; Being More Proactive

This theme had the highest number of responses. Respondents were motivated, more equipped and more determined to improve young people's transition following the conference. They had a renewed sense of vigour, felt re‐energised and empowered to drive change and make a difference.

## 4 Discussion

Healthcare transition for young people was highlighted in the NHS Long Term Plan 2019 [9], with the recommendations for a 0–25‐year healthcare service, and in 2023, results and recommendations were published by the National Confidential Enquiry into Patient Outcome and Death [10]. This enquiry undertaken between 1 October 2019 and 31 March 2021 sought to assess the barriers and facilitators for young people receiving good transition to adult healthcare services. Alongside this is the recently updated National Institute of Health and Care Excellence (NICE) Quality Standard Transition from children's to adults' services [QS140] [11]. Much has been written about transition, reports and guidance published and updated, but what we heard from delegates is that it remains a struggle to develop and implement this complex intervention.

Our unique event was organised by people who are passionate about improving young people's transition experience. Those who attended were a range of staff from health, social care and local authorities from across England, young people, those who develop national policy and voluntary, community and social enterprises. Attendance was outstanding, assisted by its hybrid nature, to an event about an issue that weighs heavily on those who care for young people. Isabel Barlow (Izzy), a young person with lived experience of transitioning into adult services, wrote ‘I was incredibly honoured to be sitting in a room with so many that had that much power to make a true difference’. We have a duty to ensure that we do not let them down. Another young person, Aishah Farooq, a Board Member on Children and Young People's (CYP) Transformation Programme Board at NHS England, emphasised the importance of young people being active participants in the conference, ‘Young person representation at the conference brought to life our unique perspectives and shifted attention to the specific needs and challenges that CYP face during what can be a time of uncertainty and significant change in their healthcare journey’.

Parallels with recent guidance was highlighted, for example, it was interesting and reassuring to observe recommendations from the NCEPOD report complement our findings [10]. This gives reassurance to those who attended to see recurring themes that already have national attention. For Question 1, the highest number of responses were about improving the transition process, up to and including developing whole services, and many suggested ways in which they could do that, for example, through developing a transition passport or putting a clear pathway in place. Some have embryonic services already – but whether developing or maintaining them, or making small changes, it was acknowledged that it requires the input, encouragement and financial support from those in management and in charge of commissioning and budgets. Small changes can be made, but creating a new infrastructure and an ethos of care requires the input, and continued interest and support of all those involved.

Communication was another key theme, by enabling people to work together we also need to ensure that they can communicate with each other. Professionals will be familiar with instances of young people visiting a different doctor, who asks them their medical history – again. Izzy tells us how ‘Young people just wanted communication between their fellow doctors and staff’. Through these discussions there was common thread that the professionals wanted to do something tangible as a start, and there was a groundswell of support to share resources and to develop a national patient transition passport together. There was a pledge to take this forward, and thus aid communication between the young person and their healthcare professionals. The number of professionals involved with each young person is huge, getting communication right is important. Education and training, such as ‘Me First’ (https://www.mefirst.org.uk/conversation-around-transition/), were highlighted as being necessary for those providing care for children and young people.

Funding was highlighted as a major obstacle. Transition is a difficult and highly complex area that requires expertise and the support of many professionals across different settings. We know that the NHS is struggling for funding in many areas, and developing transition, although a priority, is often not high on organisations' agendas due to numerous other competing priorities. Despite reference in the NHS Long Term Plan [9], to date equitable provision of funding to the level required for large‐scale quality improvement has not yet been realised. This may be partly due to economic challenges and the complexity of transition, with the requirement of funding to span across organisational boundaries. Charities are also struggling in the present time, so this often‐used channel is less available and does not provide sustainability.

It is heartening to see from the results that young people are placed firmly at the centre of all decisions, changes and commitments. Indeed, the young people who attended were inspired by the event and took heart that professionals were doing something about their experience. Haris Sultan, a Board Member of the CYP Transformation Programme at NHS England, said, ‘These discussions reinforced the value of young people's active participation in shaping the transition dialogue. Moving forward, their involvement should be prioritised, letting their insights guide the enhancement of transition experiences for young individuals’. Aishah said she, ‘Personally found that having our experience centre‐stage helped ensure that solutions are not only comprehensive but also relevant and responsive to the realities of transitioning from children's into adult services’.

Collaboration was a thread that ran through the event. Networking, working together, not just as professionals but with young people and their parents was described as core. Aishah said, ‘The most powerful way of understanding what makes a smooth healthcare transition for young people is to work in partnership with them – understand their needs, treat them as equal and involve them in decisions about their health’. Involving General Practitioners (GPs) was also deemed essential as young people with a long‐term condition will still need GP care, and the link between the hospital/hospice/community care/the GP is pivotal to success.

Izzy told us that the ‘Process of personalised care is lacking in some areas of the NHS for youth when it comes to transition’. This event helped young people to understand that work is underway and that there is a commitment to make change happen from the policy level to practice; it further enhanced professional knowledge, enabled them to network with peers, and gave them a sense of commitment to further develop transition in their workplace. Some felt emboldened to take the issues back to their managers and executive boards to move developments on. All delegates are the Transition Champions, who will continue to raise awareness. This work requires and deserves the attention from those who make the funding decisions and develop policy, and we need these Champions to continue to push for change.

This event highlighted that there is power in collaboration and in developing networks to share and grow. At the time of this conference, there was an active network of professionals who were forging ahead with implementing a QI toolkit for Transition across England [12], they also provided support and helped teams to translate guidance into everyday practice. But, as importantly, they had developed a supportive and learning network which provided peer support for those who were struggling to implement transition in their places of work. The BNTNN and network members shared resources, ideas and supported each other when things were difficult. When asked about next steps, one of the themes was that they felt ‘a responsibility to get it right’. This requires guidance, support, policy and education. The previous BNTNN fulfilled these needs, and it was clear from the results of the day that attendees felt a similar forum was still required.

To conclude, we were able to show that it is possible to bring over 400 attendees including young people, health and social care professionals, transition champions and policymakers together to inform and improve practice. This was successfully achieved through hybrid methods which were inexpensive, effective, and accessible to all. There was a large geographical reach, and the delegates were able to network, make new connections, gain support and share practice. A main outcome of bringing all of these transition champions together was a renewed commitment to continuing to progress transition services, leaving the event with renewed vigour and determination to improve healthcare transition in their workplace, acknowledging that challenges remain, yet keeping young people at the heart.

## 5 Learnings From a Multi‐Stakeholder Conference

- Success requires good organisation and information technology support, a named point of contact, the ability to respond quickly to resolving problems and people delegated to monitor the online chat who must have an understanding of the content, ensuring that everyone in the chat feels that their contribution is recognised.
- It is possible to promote inclusion, represented by attendance of over 400 attendees from a broad range of staff from varying health providers from a wide geographical spread, and including young people and policymakers.
- Equitable access is made possible. People who would usually find themselves excluded from a conference/event due to the timing of the agenda, the distance to travel or restrictions in funding were able to attend, ensuring a diverse range of views were accessed.
- Being able to involve large numbers of attendees, online and in person, enabled the formation of connections, strengthening of relationships and support for each other, with commitment to future collaborations.
- Bringing people together in person and online, creates opportunities to challenge policymakers and those responsible for commissioning and national strategy.
- Being able to empower and support young people in having their voices heard on issues important to them was not impacted by hybrid methods.

## Author Contributions

**Louise Porter:** conceptualisation, writing–original draft, writing–review and editing. **Faith Gibson:** conceptualisation, writing–original draft, writing–review and editing. **Susie Aldiss:** conceptualisation, writing–original draft, writing–review and editing. **Sue Morgan:** conceptualisation, writing–original draft, writing–review and editing. **Alexandra Stanton:** conceptualisation, writing–review and editing. **Stella Carney:** conceptualisation, writing–review and editing. **Aishah Farooq:** writing–review and editing. **Isabel Barlow:** writing–review and editing. **Haris Sultan:** writing–review and editing. **Pippa Sipanoun:** conceptualisation, writing–original draft, writing–review and editing.

## Ethics Statement

The authors declare that the research arm of this project forms part of the National Transition Evaluation Study, which has received ethical and regulatory approval (IRAS ID: 313576).

## Consent

All quotes from young people are from the co‐authors of the paper. The authors have nothing to report.

## Conflicts of Interest

The authors declare no conflicts of interest.

## Data Availability

The data that support the findings of this QI project and research are available from the corresponding author upon reasonable request.
